# When HEV infection meets organ transplantation

**DOI:** 10.1097/JS9.0000000000002860

**Published:** 2025-07-04

**Authors:** Changyi Ji, Cunquan Xiong, Luyu Wang, Hongtao Wang, Ping Huang, Mengmeng Gu, Jian Wu

**Affiliations:** a Anhui Province Key Laboratory of Immunology in Chronic Diseases, Research Center of Laboratory, School of Laboratory, Bengbu Medical University, Bengbu, China; b College of Pharmacy, Jiangsu Vocational College Medicine, Yancheng, Jiangsu, China; c Department of Clinical Laboratory, The Affiliated Suzhou Hospital of Nanjing Medical University, Suzhou Municipal Hospital, Gusu School, Nanjing Medical University, Suzhou, Jiangsu, China; d Suzhou Key Laboratory of Intelligent Critical Illness Biomarkers Translational Research, Suzhou, Jiangsu, China

**Keywords:** chronic hepatitis E, hepatitis E, immunosuppression, organ transplantation, ribavirin

## Abstract

Hepatitis E virus (HEV), a single-stranded RNA virus, is a leading cause of acute viral hepatitis globally. In recent years, the incidence of hepatitis E has increased significantly, making it a major public health concern globally. Solid organ transplant (SOT) recipients, who require long-term immunosuppressive therapy to prevent graft rejection, are particularly vulnerable to HEV infection because of their immunocompromised state. This population faces a heightened risk of developing chronic hepatitis E, which can progress to liver fibrosis and cirrhosis. Therefore, it is crucial to prioritize the monitoring of SOT recipients in clinical practice, to elucidate the associated risk factors further, and implement stringent diagnostic and preventive measures. Building on previous research, this paper comprehensively reviews the virological characteristics of HEV and the epidemiological features, clinical manifestations, diagnostic approaches, and therapeutic strategies for HEV infection in SOT recipients. The aim of this study is to provide valuable insights for effectively managing, treating, and preventing HEV infection in this high-risk population.

## Introduction

Globally, hepatitis E virus (HEV) is recognized as one of the predominant etiological agents responsible for viral hepatitis^[1]^. HEV infections exhibit a global distribution, with a particularly high prevalence in low- and middle-income countries, including regions of Asia, Africa, and the Middle East^[2-4]^. However, in special populations such as pregnant women, patients with underlying liver diseases, and those with compromised or weakened immune functions, HEV infection can lead to severe diseases or even liver failure or death^[5]^. According to estimates by the World Health Organization (WHO), HEV is responsible for approximately 20 million infections annually, resulting in around 60 000 deaths each year^[6]^. The HEV was first identified in the 1980s during a significant outbreak of non-A, non-B hepatitis among Soviet military personnel stationed in Afghanistan^[7]^. HEV is a para-enveloped virus classified within the *Orthohepevirinae* subfamily of the *Hepeviridae* family. It exhibits infectivity in non-enveloped and enveloped forms^[8,9]^. HEV retains its infectivity for up to 21 days at 37°C and up to 28 days under room temperature conditions^[10]^. There are eight HEV genotypes^[11]^. HEV-1 and HEV-2 are exclusively human-pathogenic genotypes, primarily transmitted through contaminated water sources in developing countries^[12]^. HEV-3 and HEV-4 are zoonotic genotypes predominantly transmitted in Europe, with a broad host range including wild boars, deer, camels, and other animal species^[13]^. HEV-5 and HEV-6 are exclusively detected in wild boars, while HEV-7 and HEV-8 are specifically associated with camels^[2,14]^.

The extensive implementation of organ transplantation surgeries has significantly improved the quality of life of patients with end-stage organ failure. However, the immunosuppressive status after transplantation makes patients susceptible to opportunistic infections. In recent years, HEV infection has emerged as a significant cause of viral hepatitis among solid organ transplantation (SOT) recipients. Following organ transplantation, patients require long-term immunosuppressive therapy, resulting in a sustained immunosuppressed state. This immunosuppression compromises the host’s antiviral defense mechanisms, facilitating viral replication and persistence. In 2008, Kamar *et al* reported that 14 out of 217 SOT recipients were diagnosed with HEV infection, of whom 8 (57%) progressed to chronic HEV infection^[15]^. In immunocompetent individuals, HEV infection is typically asymptomatic or associated with mild, self-limiting clinical manifestations. In contrast, immunocompromised populations, including organ transplant recipients, HIV-infected individuals, and patients with hematological malignancies, are at an increased risk of developing chronic hepatitis E following HEV infection^[8,16]^.

At present, there are still deficiencies in the understanding of HEV in the field of transplant surgery: First, there is no international consensus on HEV screening during the organ acquisition process, and there is a potential risk of transmission; second, the molecular interaction mechanism between immunosuppressants and HEV replication has not been fully clarified. Furthermore, the monitoring program after transplantation lacks precise thresholds for the dynamic association between viral load and immune status. This review aims to systematically elaborate on the epidemiological characteristics, diagnostic methods, and clinical management challenges of HEV infection in organ transplant recipients. The goal is to provide a new perspective for the standardized prevention and treatment of HEV infection in organ transplant recipients^[17]^.

## Epidemiological characteristic of HEV infection in SOT patients

Hepatitis E is caused by the HEV which is a public health issue of global concern. Each year, 20 million people are infected with HEV, among which there are 3.3 million symptomatic cases and approximately 60 000 deaths, accounting for 3.3% of viral hepatitis-related deaths^[18]^. Current research shows that genotypes 1 and 2 of HEV only infect humans and are mainly transmitted through contaminated water^[5]^. Type 3 and type 4 HEV can infect humans, pigs, and other mammals. They are zoonotic diseases and mainly cause sporadic cases^[19]^. The majority of chronic hepatitis E cases discovered so far are caused by genotype 3 HEV^[15]^.

SOT is a surgical procedure that involves the removal of functional organs (such as the heart, liver, and kidneys) to replace damaged or failed organs within a patient’s body. This intervention aims to enhance the survival rate of individuals suffering from organ failure^[20]^. However, organ transplant recipients, who require immunosuppressive therapy to mitigate the risk of organ rejection post-surgery, are at an elevated risk for HEV infection^[21]^. The infection rate of HEV among organ transplant recipients has been reported to range from 1% to 2%. Furthermore, over 60% of SOT recipients who are infected with HEV will progress to develop chronic hepatitis E^[22]^. This condition is characterized by the persistence of HEV RNA in the patient’s serum, stool, and liver biopsy samples for more than 3 months^[8,15]^.

In a meta-analysis encompassing 20 studies with a total of 5842 patients, the prevalence of HEV infection among SOT recipients was found to be 20.2%. This prevalence varied by organ type: liver transplants exhibited an infection rate of 27.2%, while kidney and heart transplants both had rates of 12.8%. In contrast, lung transplant recipients demonstrated a significantly lower prevalence at just 5.6%. Furthermore, it is noteworthy that the incidence of HEV infection in middle-income countries is twice as high compared to that in high-income countries^[23]^. In 2008, French researchers, Kamar *et al*, reported that among 217 patients who underwent SOT including 3 liver recipients, 9 kidney recipients, and 2 combined kidney and pancreas transplant recipients-14 tested positive for HEV RNA. Subsequent follow-up revealed that of these cases, six developed acute HEV infection while eight progressed to chronic HEV infection. This latter group included three individuals with liver transplants, three with kidney transplants, and two who received combined pancreas–kidney transplants. Additionally, liver tissue biopsies indicated that nine patients from the kidney transplant cohort, three from the liver transplant cohort, and one from the combined kidney–pancreas transplant group developed liver fibrosis^[15]^.

**HIGHLIGHTS**

- HEV infection has emerged as a significant cause of viral hepatitis among solid organ transplantation (SOT) recipients.
- The population faces a heightened risk of developing chronic hepatitis E, which can progress to liver fibrosis and cirrhosis.
- It provides valuable insights for effectively managing, treating, and preventing HEV infection in this high-risk population.

In 2011, French scholars, Legrand-Abravanel *et al*^[24]^ evaluated the HEV prevalence rate in 499 transplant recipients (376 kidney transplant recipients and 123 liver transplant recipients). With a median follow-up period of 22 months, a total of 34 cases of HEV infection were identified, comprising 22 in kidney transplant recipients and 12 in liver transplant recipients. Among these patients, 16 developed chronic HEV infection, while 18 experienced viremia that resolved following acute infection, with no recurrence or reinfection observed during the follow-up period. In 2012, Pischke *et al* reported on HEV infection among 274 heart transplant recipients, alongside a cohort of 137 non-transplant heart patients who underwent cardiac surgery, and 537 healthy individuals recruited as a control group. Among the 274 heart transplant recipients, 31 (11.3%) tested positive for anti-HEV antibodies, while 4 (1.5%) were found to be HEV RNA positive. In the group of non-transplant heart patients, anti-HEV antibodies were detected in 10 (7.3%), with no patients testing positive for HEV RNA. Within the healthy population, anti-HEV antibodies were identified in 11 individuals (2.0%), and none exhibited positivity for HEV RNA^[25]^. The study revealed that the prevalence of HEV antibodies among heart transplant recipients and non-transplant patients undergoing cardiac surgery was four to five times greater than that observed in healthy individuals. This increased prevalence is attributed to an increased risk of HEV exposure, which is associated with the more frequent use of blood products in patients suffering from heart disease.

In 2013, Dutch researchers, Riezebos-Brilman *et al*, found that among 468 adult lung transplant recipients, 10 patients (2.1%) tested positive for HEV RNA, all of whom were infected with genotype 3 strains^[26]^. At the time of HEV testing, all patients exhibited elevated liver enzymes, with a median alanine aminotransferase level of 77 U/L, and presented with mild hepatitis. Eight lung transplant recipients were diagnosed with developed nosed with chronic HEV infection following transplantation. Additionally, two patients who received oral ribavirin (RBV) at a dosage of 400 mg twice daily underwent treatment for acute HEV infection after 2 months when serum HEV RNA became undetectable and liver aminotransferase levels normalized. In 2014, Abravanel *et al*^[27]^, from France screened 263 SOT recipients for anti-HEV immunoglobulin G (IgG) antibodies. The results showed that 38.4% of the participants tested positive for anti-HEV IgG at the time of transplantation. During the 1-year follow-up period, three new cases of HEV infection and three instances of reinfection were documented; notably, one case of reinfection became chronic.

A study in 2024 found transplant rejection (OR = 0.075; *P* = 0.045) was negatively correlated with serum-negative transformation in patients with positive anti-HEV antibodies before transplantation^[28]^. However, studies on the clinical effects of HEV infection on SOT recipients, such as transplantation failure, rejection, and mortality, are still insufficient. There is an urgent need for multicenter studies with larger scale, more cases, and longer follow-up.

In summary, HEV infection is common among SOT recipients. Notably, the incidence of HEV infection is much higher in liver transplant recipients compared to those receiving other types of organ transplants. These epidemiological features suggest a high risk of HEV infection (Table 1), while its clinical manifestations further highlight the unique course of the disease in immunosuppressed patients.

**Table 1:** Cases of organ transplant patients infected with HEV in the last 10 years

Organ type	Year	Country	Detection method	Caseload	Reference
Liver	2017	America	HEV IgG	15	^[29]^
	2018	America	IgM	12	^[30]^
	2018	Greece	HEV RNA	1	^[31]^
	2019	Croatia	HEVIgG/IgM/RNA	59	^[32]^
	2020	Germany	HEV IgG or HEV RNA	21	^[33]^
	2020	Italy	HEVIgG/IgM/RNA	23	^[34]^
	2020	Thailand	HEV IgG/IgM/RNA	48	^[35]^
	2021	Turkey	HEV IgG and IgM	31	^[36]^
	2022	Turkey	HEV IgG	59	^[37]^
	2024	Thailand	HEV IgG/IgM/RNA	27	^[38]^
	2024	China	HEV IgG/IgM/RNA	65	^[28]^
Kidney	2015	France	HEV IgG	75	^[39]^
	2015	Iran	HEV IgG and IgM	19	^[40]^
	2015	Italy	HEV IgG and IgM	4	^[41]^
	2017	America	HEV IgG	19	^[29]^
	2018	Netherlands	HEV RNA	2	^[42]^
	2018	America	HEV IgM	2	^[30]^
	2018	Germany	HEV IgG and IgM	12	^[43]^
	2019	Japan	HEV IgG	8	^[44]^
	2020	Germany	HEV IgG/IgM/RNA	59	^[45]^
	2020	Japan	HEV IgG	103	^[46]^
	2022	Turkey	HEV IgG	25	^[37]^
	2023	Thailand	HEV IgG	15	^[47]^
Heart	2015	Sweden	HEV RNA	1	^[48]^
	2018	America	HEV IgM	1	^[30]^
	2019	France	HEV IgG and IgM	4	^[49]^
	2020	Japan	HEV IgG	7	^[46]^
Lung	2015	Sweden	Ig G	8	^[50]^
	2019	Spain	HEV IgG/IgM/RNA	3	^[51]^

## Clinical manifestations of HEV infection in SOT patients

HEV infection usually presents as an asymptomatic or self-limiting acute hepatitis in immunocompetent individuals, with a disease course generally persisting for several weeks. Still, after organ transplant recipients are infected with HEV, they usually cannot clear HEV from their bodies in the short-term relying on their immunity and are prone to develop chronic HEV infection^[52]^.

The clinical presentation of HEV infection is very similar to hepatitis A virus infection, characterized by elevated hepatic transaminases (alanine transaminase and aspartate transaminase), constitutional symptoms including fatigue and myalgia, gastrointestinal manifestations such as abdominal pain, nausea, and vomiting, as well as potential jaundice development^[12]^. The liver serves as the principal site for HEV replication in vivo; however, recent research has shown that HEV infection is associated with a spectrum of extrahepatic manifestations across multiple organ systems. These systemic complications include pancreatic involvement (pancreatitis), hematological disorders (particularly anemia), renal impairment, neurological sequelae (including Guillain-Barré syndrome and meningoencephalitis), reproductive system abnormalities (notably male infertility), and immune-mediated hepatic injury (autoimmune hepatitis)^[12,53]^. Studies have found that neurological manifestations are more common in HEV-infected patients with normal immune function^[54]^. Van found that 10.6% of the patients suffered from acute hepatitis E and all had bilateral brachial plexus nerve involvement manifestations^[55]^.

Following HEV infection, SOT recipients typically demonstrate impaired viral clearance capacity due to immunosuppressive therapy, rendering them particularly susceptible to developing chronic HEV infection. The clinical spectrum of chronic hepatitis E includes both persistent symptoms resembling acute infection and progressive hepatic parenchymal injury, which may ultimately lead to liver fibrosis and cirrhosis if left untreated. In a seminal case report, Haagsma *et a.*, a research group from the Netherlands, first documented two instances of chronic HEV infection in liver transplant recipients, which marked a significant milestone in the understanding of HEV pathogenesis in immunocompromised patients^[56]^. Patient A developed idiopathic chronic hepatitis 2 months post-liver transplantation, with subsequent progression to hepatic cirrhosis that required 7 years following the initial procedure. In contrast, Patient B presented with cryptogenic chronic hepatitis 7 years posttransplantation, requiring retransplantation after a 5-year interval; notably, this patient remained free of recurrent hepatitis symptoms following the second transplantation. Pischke *et al* published a pivotal case series documenting three instances of HEV infection in post-liver transplantation patients, significantly contributing to the understanding of HEV epidemiology in immunosuppressed populations^[57]^. Among these cases, two patients developed chronic HEV infection, while one patient developed advanced hepatic fibrosis within 22 months following acute infection. In a notable 2012 Canadian case study, researchers documented a pediatric patient who remained seropositive for both anti-HEV IgG and immunoglobulin M (IgM) 8 years posttransplantation. This case was particularly remarkable as HEV RNA remained detectable through annual monitoring over a decade-long period posttransplantation, with sustained elevation of hepatic transaminases and eventual progression to cirrhosis^[58]^. In the context of organ transplantation, a subset of recipients experiences transient elevation of hepatic transaminases shortly after transplantation and patients with chronic HEV infection typically exhibit persistent or fluctuating abnormalities in liver function tests, reflecting ongoing hepatic inflammation and potential parenchymal injury^[8]^.

Furthermore, the risk of chronic and severe cases is relatively low in the general population (i.e., those with normal immunity), and extrahepatic manifestations are rare. Immunosuppressed populations may involve the nervous system. A representative case from France involved a renal transplant recipient with chronic HEV infection who presented with a complex neurological syndrome characterized by peripheral neuropathy manifesting as proximal muscle weakness, central nervous system involvement affecting limb coordination, and bilateral pyramidal tract signs. Notably, virological analysis confirmed the presence of HEV RNA in both serum and cerebrospinal fluid, demonstrating the neurotropic potential of the virus^[59]^.

## Serum and etiological diagnosis of HEV infection in SOT patients

Serum anti-HEV IgG and IgM were detected by ELISA^[60]^. The serological profile of HEV infection exhibits distinct temporal dynamics: anti-HEV IgM antibodies appear during the acute phase of infection and generally persist for approximately 3 months, whereas anti-HEV IgG antibodies, indicative of prior exposure, remain detectable for more than a decade post-infection. This differential antibody kinetics offers critical diagnostic utility in distinguishing between acute and previous HEV infections, aiding in the accurate assessment of infection status and timing^[61]^. HEV RNA is detectable in both serum and fecal samples before the onset of clinical symptoms, with fecal specimens exhibiting a significantly extended duration of detectability compared to serum. This prolonged presence of viral RNA in feces underscores its utility as a sensitive marker for early infection and ongoing viral shedding^[7,62,63]^. In patients with acute HEV infection, both anti-HEV IgG and IgM antibodies are usually detectable. However, the immunocompromised state of most organ transplant recipients often leads to negative results in serological tests for anti-HEV IgG and IgM. Consequently, direct detection of HEV antigen and viral RNA in blood or fecal samples is crucial for the accurate diagnosis of HEV infection in this population^[64–66]^.

In a landmark 10-year multicenter study conducted from July 2013 to June 2023, Chiu *et al* performed a retrospective analysis encompassing all adult patients who underwent HEV IgM serological testing or HEV RNA detection at Mayo Clinic facilities. This extensive investigation provides critical insights into the epidemiology and diagnostic patterns of HEV infection over a decade^[67]^. The study cohort included 1640 patients who were tested for either HEV IgM antibodies or HEV RNA. The distribution of testing revealed that 1293 patients (79%) underwent HEV IgM testing alone, 213 patients (13%) were tested exclusively for HEV RNA, and 134 patients (8%) received both HEV IgM and HEV RNA testing. Among these cases, 18 HEV infections were identified, comprising 16 acute infections and 2 chronic infections. The two-step IgM antibody testing protocol, which initially uses the recomWell HEV IgM ELISA followed by the recomLine HEV IgM Strip test for positive or equivocal samples, showed limitations in detecting chronic HEV infections. Specifically, this diagnostic approach failed to identify two cases of chronic HEV infection in SOT recipients. However, the two-step HEV IgM antibody testing protocol proved to be a reliable diagnostic tool for acute HEV infection in immunocompetent individuals. However, this diagnostic approach demonstrates notable limitations in detecting chronic infections, which require HEV RNA detection for precise diagnosis. As outlined in the clinical practice guidelines by the European Association for the Study of the Liver (EASL), nucleic acid testing is the recommended diagnostic method for chronic HEV infection^[7]^.

Urine sample collection is simple and painless, avoiding the discomfort and risks of blood collection. It is suitable for mass screening, especially in epidemic outbreaks or high-incidence areas, to quickly identify infected people and control transmission. HEV antigen is a pivotal biomarker for the detection of hepatitis E infection. Its diagnostic value is amplified by its specific uptake by renal tubular epithelial cells and subsequent excretion into urine. This physiological concentration mechanism leads to approximately 10-fold higher antigen levels in urine than in serum, thereby endowing urinary antigen testing with greater diagnostic sensitivity compared to traditional serum antibody detection methods^[68,69]^. Approximately one-third of individuals infected with HEV achieve spontaneous viral clearance. As a result, current clinical guidelines advocate for molecular testing at 3 months post-diagnosis to assess whether viral persistence or clearance has occurred. Additionally, quantitative molecular assays are invaluable for monitoring and evaluating the effectiveness of therapeutic interventions in HEV treatment protocols.

To sum up, HEV infection after organ transplantation may present insidious manifestations, and the test results of anti-HEV antibodies in some patients may be negative, leading to an increased risk of missed diagnosis in clinical practice^[70]^. Therefore, for patients with abnormal liver function after SOT, clinicians should be highly vigilant about the possibility of HEV infection and conduct HEV antibody and nucleic acid tests promptly for a clear diagnosis.

## Treatment of HEV infection in SOT patients

**Figure 1.**
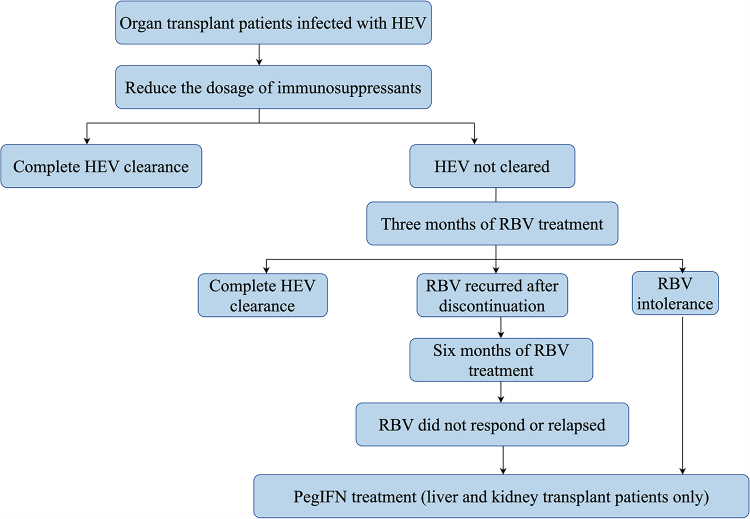
Flowchart of HEV infection treatment for solid organ transplant patients.

HEV: hepatitis E virus; RBV: ribavirin; PegIFN: pegylated interferon.

In the general population, HEV infection is typically self-limiting and resolves spontaneously. Usually, treatment measures are only taken for patients with severe symptoms, and there are no specific treatment drugs or methods yet. However, in European countries, approximately two-thirds of SOT recipients infected with HEV progress to chronic hepatitis E, highlighting the significant impact of immunosuppression on disease progression in this vulnerable population^[8]^. Within 3–5 years, nearly 10% of SOT patients infected with HEV will develop cirrhosis^[71]^. For organ transplant recipients, a subset of patients may achieve viral clearance by reducing their immunosuppressive therapy. Should this approach prove ineffective, initiating antiviral treatment should be considered a subsequent therapeutic strategy (Figure 1)^[72]^.

### Immunosuppressant

A multicenter study published by the EASL reveals that reducing the dosage of immunosuppressive drugs can result in sustained viral clearance in approximately 30% of transplant recipients with chronic HEV infections. This finding underscores the potential of immunosuppression modulation as a therapeutic strategy in this patient population^[7]^. HEV infection affects more than 60% of SOT recipients, frequently leading to chronic hepatitis. Tacrolimus has been recognized as a major risk factor for the progression to chronic hepatitis E in this population^[15]^, which is an immunosuppressive drug used to prevent and treat organ rejection in liver, heart, and kidney transplant recipients^[73–75]^. Furthermore, tacrolimus specifically disrupts T-cell signal transduction and activation by inhibiting the transcription and release of cytokines^[22]^. It also suppresses the adaptive immune response to the virus by suppressing T-cell activation. In a retrospective analysis encompassing organ transplant recipients from 17 centers across Europe and the United States, Kamar *et al* examined the factors contributing to the progression of chronic HEV infection in this cohort^[22]^. The study included a total of 85 transplant recipients, of whom 56 developed chronic HEV infections. Among these, 18 patients (32.1%) achieved HEV clearance after a reduction in their immunosuppressive therapy dosage. Furthermore, 20 patients attained HEV clearance following antiviral treatment. At the final follow-up, 14 individuals had successfully cleared the virus, while 6 remained under ongoing treatment.

Among the remaining 18 patients who did not receive antiviral therapy, 5 remained HEV RNA-positive in serum and ultimately succumbed to various complications: 2 from decompensated cirrhosis, 1 from septic shock secondary to a liver abscess, 1 due to acute respiratory distress syndrome, and 1 from hepatocellular carcinoma. The other 13 patients continue to harbor HEV infection. However, reducing immunosuppression is not a universally applicable strategy for all transplant recipients, as it may elevate the risk of acute rejection, particularly in those who have undergone heart, lung, or pancreas transplantation^[8]^. Emerging studies have demonstrated that T lymphocytes play a pivotal role in the clearance of HEV, highlighting their critical contribution to the immune response against HEV infection^[15,76]^. Therefore, under safe circumstances, it is recommended to try to reduce the dosage of immunosuppressants or adjust the use regimen of immunosuppressants, while maintaining sufficient T lymphocyte levels to ensure virus clearance and graft tolerance.

### Antiviral therapy

RBV (C_8_H_12_N_4_O_5_), a purine nucleoside analog with broad-spectrum antiviral activity, is the mainstay treatment for chronic HEV infection in transplant recipients, despite the absence of formal regulatory approval for this specific indication^[77]^. Despite the lack of approved therapeutic protocols for chronic HEV infection, RBV has established itself as the first-line antiviral treatment. Clinical evidence supporting its efficacy includes a prospective study of 59 SOT recipients, where a 3-month RBV regimen achieved a sustained virological response (SVR) in 78% (46/59) of patients. Notably, among those who experienced virological relapse, retreatment with RBV for 6 months successfully achieved SVR, highlighting its therapeutic potential in managing refractory cases^[78]^. Pischke *et al* evaluated the therapeutic efficacy of RBV by analyzing the clinical progression of HEV infection in 15 organ transplant recipients^[79]^. Eleven of these patients were treated with RBV, and nine demonstrated a significant response, achieving undetectable serum HEV-RNA levels.

In a separate study, Kamar *et al* compiled data from 30 centers across Europe and analyzed information from 255 organ transplant recipients to evaluate the efficacy of RBVin the treatment of chronic HEV infection^[80]^. The median dose of RBV administered was 600 mg/day (range: 29–1200 mg/day), with a mean dose of 8.6 ± 3.6 mg/kg/day. The median treatment duration was 3 months (range: 0.25–18 months). After the initial RBV treatment, the SVR rate was 81.2%. Notably, this SVR rate increased to 89.8% in some patients following a second course of treatment; however, approximately 28% of patients required dose adjustments due to RBV-related adverse effects. Common side effects included hemolytic anemia, mood disorders, and sleep disturbances^[81,82]^. Current guidelines for RBV use are largely extrapolated from clinical experience with hepatitis C (HCV). Since RBV is primarily excreted through the kidneys, patients with renal insufficiency face a higher risk of drug accumulation. This accumulation can exacerbate side effects, necessitating dosage adjustments based on renal function^[83]^. Additionally, RBV is recognized for its teratogenic potential, presenting significant risks to pregnant women and their developing fetuses^[84]^. Therefore, RBV should be used with caution, especially in special populations.

RBV is currently the only treatment option for many patients, but treatment failure may be related to the emergence of a unique HEV polymerase mutant (G1634R)^[85]^. A study included 12 patients with chronic HEV genotype 3 infection, 9 patients received RBV treatment, among whom 5 successfully cleared HEV and 4 failed the treatment^[86]^. After detecting the variations of the HEV genome using Illumina deep sequencing technology, it was found that among the patients who failed the treatment, the G1634R mutation existed as a small group before the treatment and gradually increased during the RBV treatment, becoming the dominant group. G1634R may be used to identify patients at risk of treatment failure at an early stage, facilitating doctors to adjust treatment strategies. The Y1320H mutation in the RNA-dependent RNA polymerase (RdRp) of the HEV may also contribute to RBV treatment failure^[87]^. The Y1320H and G1634R mutations in the RdRp domain promote antiviral resistance by enhancing HEV replication^[88]^. However, at present, there is a lack of clinical studies on the G1634R/Y1320H mutation, such as the drug resistance rate. These studies provide a substantial foundation for the use of RBV in treating chronic HEV infection in organ transplant recipients. However, it is important to note that these studies lacked a control group receiving RBV, and the specific dosage of RBV was not clearly defined. Consequently, further research is urgently) needed to establish the optimal RBV dosage to ensure its safe and effective application.

Pegylated interferon (PegIFN) was first used in the treatment of chronic hepatitis C in the late 20th century. Over time, its application has gradually expanded to include the treatment of chronic hepatitis B^[89,90]^. Organ transplant patients, particularly liver and kidney transplant recipients, who are intolerant to RBV may be treated with PegIFN. Ollivier-Hourmand documented a case of a kidney transplant recipient who developed resistance to RBV therapy and subsequently achieved an SVR 10 months after initiating Peg-IFN treatment^[91]^. Kamar, a French researcher, included three patients with chronic active HEV infection following liver transplantation in his study^[92]^. The patients were treated with PegIFN alpha (Peg-IFN-α) for 3 months. After treatment, two patients achieved an SVR at 6 and 5 months, respectively, demonstrating significant and lasting reductions in their viral loads. In contrast, the third patient experienced a relapse.

In the clinical use of antiviral drugs for HEV, especially PegIFN, it is crucial to monitor the immune status of organ transplant recipients closely. Additionally, timely adjustments to the dosage of antirejection medications are necessary to ensure patient safety. Therefore, the initial management strategy should take patient safety as the primary principle and prioritize reducing the dosage of immunosuppressants. Approximately 30% of patients can achieve spontaneous clearance of HEV through this approach^[93]^. If the virus persists, RBV monotherapy can be used for 3 months and if the treatment fails again, RBV treatment will continue for 6 months^[7]^. For liver and kidney transplant patients, PEG-IFN-α treatment can be considered, but there are no clear recommendations for other SOT patients for the time being.

### New antiviral drugs

**Figure 2.**
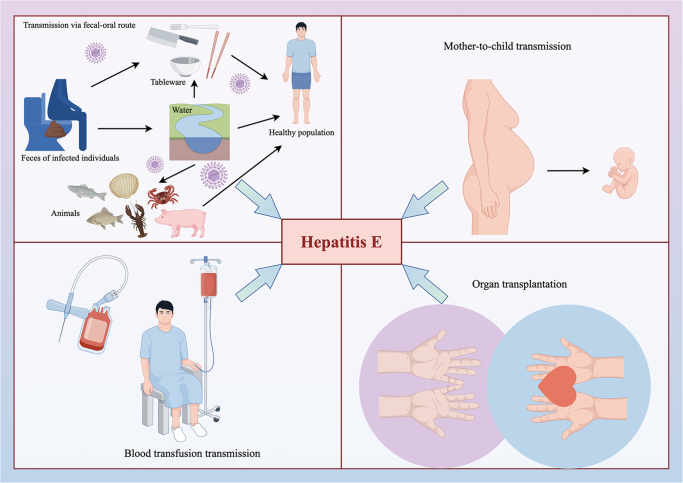
The transmission routes of the hepatitis E virus.

Developing new antiviral drugs has enriched the treatment strategies for hepatitis E. In recent years, significant progress has been made in the research and development of antiviral medications for HEV, but its safety and antiviral efficacy still need to be confirmed by a large number of clinical trials. Bhise *et al* discovered that the antimalarial drug artemisinin (ART) can impede the replication of HEV by targeting two nonstructural proteins (helicase and RdRP) and the inhibition rates of HEV-1 and HEV-3 were 59% and 43% respectively^[94]^. Moreover, ART is safe for pregnant women. Favipiravir is an FDA-approved RdRp inhibitor and is currently used to treat coronavirus, HCV, and influenza virus infections^[95–97]^. Hooda *et al* found that the combined use of favipiravir and sofosbuvir produced additional antiviral effects^[98]^. Kaushik *et al* found that zinc salts inhibited the replication of genotype 1 (g-1) and g-3 HEV replicators and infectious genomic RNA of g-1 HEV in a dose-dependent manner. Zinc salts directly inhibit the activity of viral RdRp, thereby suppressing viral replication^[99]^.According to expert consensus and established guidelines, the treatment protocol for HEV infection in patients who have undergone SOT is illustrated in Figure 2. The analysis of the advantages and disadvantages of therapeutic drugs for SOT patients infected with HEV is shown in Table 2.

The Hepatitis E virus can be transmitted through the fecal-oral route, mother-to-child transmission, blood transfusion transmission, and organ transplantation route.

**Table 2:** Treatment of HEV infection in SOT patients

Treatment method	Advantages	Disadvantages	Mechanism
Immunosuppressant	This contributes to the restoration of immune function and facilitates the clearance of the virus.	May elevate the risk of organ rejection, necessitating close monitoring.	This facilitates the restoration of immune system functionality, thereby enhancing the capacity to eliminate HEV.
Ribavirin	It is effective for most HEV-infected individuals, demonstrating a high viral clearance rate.	It may induce adverse effects such as hemolytic anemia, among others.	It inhibits HEV replication by targeting the viral RNA-dependent RNA polymerase.
Pegylated interferon	It exhibits direct antiviral activity and possesses immunomodulatory effects.	It is associated with a range of adverse effects, including flu-like symptoms and bone marrow suppression, among others.	Interferon activates intracellular antiviral proteins, thereby inhibiting HEV replication. It enhances the activity of natural killer cells and T cells, thereby promoting viral clearance.

## The immunological mechanism of HEV infection in SOT patients

The immune response is a key factor determining the clinical manifestations and outcomes of patients with hepatitis E. A normal immune response is conducive to virus clearance and disease recovery, while an excessive or disordered immune response can be involved in the process of its severe and chronic development, leading to disease progression.

Studies have shown that in SOT patients infected with HEV, the proportions of various immune cells have decreased to varying degrees. Specifically, the proportion of natural killer (NK) cells in the peripheral blood of these patients was significantly lower than that of SOT patients who were not infected with HEV^[100]^. Interferon-gamma (IFN-γ) secreted by NK cells plays a key role in resisting HEV infection. However, in SOT patients treated with immunosuppressants, the level of IFN-γ is also decreased, resulting in a reduction of downstream inflammatory factors and insufficient activation of adaptive immune cells^[101]^, which might be one of the reasons for the chronic transformation of hepatitis E. Immunosuppressed patients infected with HEV may present with T cell exhaustion, which may be related to the progression of chronic diseases. Kemming *et al* found that chronic HEV infection is related to the depletion of HEV-specific CD8+ T cells^[102]^, and virus escape can lead to the exhaustion of T cells.

## Preventing HEV infection in SOT patients

SOT recipients exhibit an increased susceptibility to HEV infection, a risk that is further exacerbated by the extensive use of immunosuppressive agents. It is crucial for these patients to follow their healthcare provider’s guidance on the prudent administration of such medications and to undergo consistent surveillance of their immunological parameters^[8]^. This patient population is also strongly advised to avoid consuming inadequately cooked meat and seafood. The administration of large volumes of blood products, plasmapheresis, and stem cell transplantation are procedures that significantly augment the risk of HEV infection via the hematogenous route^[103]^. In scenarios where blood transfusion is indispensable, it is essential to prioritize the safety of blood products by opting for those that have been subjected to stringent screening and testing protocols to minimize the potential for transfusion-transmitted HEV^[104]^. Moreover, as a preventive measure, both donors and recipients should be evaluated for HEV before transplantation. In alignment with these precautions, the EASL advocates for HEV screening in all immunocompromised patients who present with liver enzyme elevations of unknown etiology^[7]^.

In addition, vaccination against hepatitis E is an important strategy to reduce the burden of the HEV. Effective vaccines can prevent symptomatic HEV infection in susceptible populations. The recombinant hepatitis E vaccine jointly developed by Xiamen University and Xiamen Wantai Canghai Biotechnology Co., Ltd. has been approved for sale and use in the Chinese market. The results of phase I–III clinical trials of the HEV 239 vaccine indicated that the vaccine had favorable safety, efficacy, and immunogenicity profiles in healthy volunteers aged 16−65^[105,106]^. The WHO endorsed the hepatitis E vaccine in its 2015 position paper during the outbreak of hepatitis E which is expected to benefit more high-risk populations by using this vaccine^[107]^.

## Problems and challenges

### Diagnostic difficulty

In immunocompromised organ transplant recipients, HEV infection often manifests with atypical symptoms, such as mild fatigue or even complete absence of clinical signs, posing significant challenges to early diagnosis. The immunosuppressed state in these patients can impair their ability to mount a robust antibody response, leading to potential false-negative results in serological assays, including anti-HEV IgM and IgG testing. As a result, confirmatory diagnosis necessitates the detection of HEV RNA through polymerase chain reaction to ensure accuracy. Additionally, the clinical presentation of HEV infection may closely mimic other prevalent conditions in this population, such as drug-induced liver injury and transplant-related complications, further complicating the diagnostic process. This overlap underscores the importance of a comprehensive diagnostic approach to differentiate HEV infection from other etiologies of liver dysfunction in this vulnerable cohort^[108,109]^.

## Limited treatment options

At present, there are no targeted antiviral therapies approved for the treatment of HEV infection. While RBV has shown potential efficacy in some clinical studies, critical aspects such as the optimal dosing regimen, treatment duration, and reliable predictors of therapeutic response remain poorly defined. Further research is needed to establish standardized guidelines for the use of RBV in managing HEV infection, particularly in immunocompromised populations^[78,110]^. The reduction of immunosuppressive therapy is considered the primary strategy for managing chronic HEV infection. However, an excessive decrease in immunosuppression may precipitate organ rejection in transplant recipients, underscoring the necessity of achieving a delicate balance between suppressing viral replication and maintaining graft tolerance. Furthermore, the use of RBV, a commonly employed antiviral agent, is often limited by its associated adverse effects, including hemolytic anemia and neuropsychiatric disturbances, which can significantly compromise treatment adherence and diminish patients’ quality of life. These challenges highlight the need for a tailored therapeutic approach that optimizes both viral control and patient well-being^[111]^.

## The complexity of immune states

Organ transplant recipients require long-term use of immunosuppressants, which not only increases the risk of HEV infection but may also affect viral clearance and response to treatment. Significant differences in immune status and response to immunosuppressants in different patients complicate individualized adjustment of treatment regimens.

## Monitoring and management

The management of HEV infection is often prolonged, requiring consistent monitoring of viral load, liver function parameters, and drug levels. This process places substantial demands on healthcare resources and challenges patient adherence to treatment regimens. Even when viral load reduction is achieved during therapy, a subset of patients may experience relapse, necessitating further therapeutic interventions and extended follow-up to ensure sustained viral control and prevent complications.

## Clinical communication and science popularization

The management of HEV infection requires the collaboration of a multidisciplinary team of transplant physicians, infectious disease specialists, hepatologists, and clinical pharmacists to provide comprehensive medical support. Regular publicity campaigns for hepatitis E in hospitals can not only improve patients’ understanding of the disease but also prompt high-risk groups to undergo screening and treatment. At the same time, confirmed patients can also better avoid high-risk behaviors through popular science education to effectively control the spread of the disease. In addition, popular science education can also enhance patients’ confidence in treatment, improve their enthusiasm for treatment, and thus achieve better treatment effects and effectively curb the spread of hepatitis E.

## Prospect

Organ transplantation is a major advance in modern medicine, bringing hope to many patients with end-stage organ failure. However, the immunosuppressed state that patients face after transplantation makes them more susceptible to various pathogens, of which HEV infection is of particular concern. The vast majority of HEV infections can be asymptomatic or mild symptoms. With the deepening of HEV research, humans have recognized that hepatitis E is not only a self-limited disease. HEV infection in organ transplant patients may be prolonged to chronic infection or even cirrhosis, which is a serious threat to the life and health of patients.

Although some progress has been made in the diagnosis and treatment of HEV in recent years, many challenges remain. First, the clinical manifestations of HEV infection are diverse, and symptoms are often atypical in immunocompromised patients, which increases the difficulty of early diagnosis. Second, there are currently limited treatment options for HEV infection, and RBV has shown good efficacy in some patients, but its optimal dose, duration of treatment, and how to effectively monitor treatment response remain to be further studied. In addition, the side effects of RBV, such as hemolytic anemia and mood disorders, also pose a challenge to patient compliance.

Future research directions should include the development of more sensitive and specific diagnostic tools for early detection and accurate diagnosis of HEV infection. At the same time, new antiviral drugs and treatment strategies need to be further explored to improve treatment effectiveness and reduce side effects. In addition, more attention should be paid to prevention measures for HEV infection, such as reducing the risk of HEV transmission by improving public health conditions, strengthening food safety supervision, and developing effective vaccines.

In clinical practice, the collaboration of multidisciplinary teams is essential. Transplant doctors, infectious disease specialists, hepatologists, and clinical pharmacists should work together to provide patients with comprehensive guidelines for diagnosing and treating HEV infection after organ transplantation to further improve transplant success and long-term survival.

## Data Availability

Not applicable.
